# Vascular Leiomyosarcoma Masquerading as Chronic Deep Venous Thrombosis

**DOI:** 10.1097/RLU.0000000000006240

**Published:** 2025-12-11

**Authors:** Isabella Phillips, Jason Chen, Mohammed Milhem, Paul DiCamillo, Janet Pollard

**Affiliations:** * Department of Internal Medicine, University of Iowa Carver College of Medicine; † Department of Internal Medicine, Holden Comprehensive Cancer Center, University of Iowa Health Care; ‡ Department of Radiology, University of Iowa Health Care, Iowa City, IA

**Keywords:** leiomyosarcoma, intravascular, metastasis

## Abstract

A 78-year-old man with a history of DVT on apixaban presented with bilateral leg pain and dyspnea. Chest CTA ruled out pulmonary embolism but revealed bilateral pulmonary nodules. Biopsy identified a spindle cell neoplasm. ^18^F-FDG PET and duplex ultrasound imaging revealed intensely FDG-avid, intraluminal masses in the right femoral and popliteal veins. MRI confirmed lesions with peripheral enhancement. Biopsy of one of the femoral vein masses showed a high-grade spindle cell neoplasm positive for SMSA and desmin, consistent with leiomyosarcoma. This rare vascular tumor mimicked DVT, highlighting the importance of multimodal imaging and biopsy in diagnosing intravascular malignancies with metastatic potential.

**FIGURE 1:**
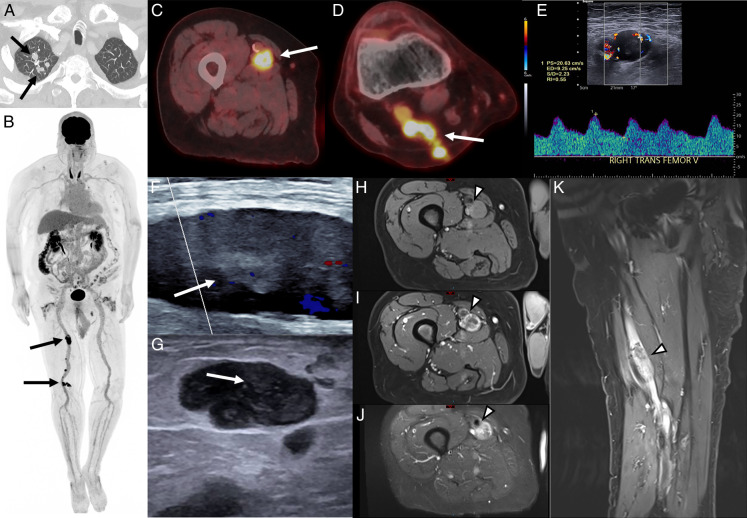
A 78-year-old man with a history of deep vein thrombosis (DVT) and pulmonary embolism on apixaban presented with dyspnea and 2–3 weeks of bilateral leg pain. A duplex ultrasound diagnosed acute DVT, apparently without consideration of tumor as a differential (not shown). A chest CT angiogram ruled out pulmonary embolism but incidentally identified multiple noncalcified upper lobe predominant nodules (**A**). The differential diagnosis included infection versus metastatic malignancy. Bronchoscopic biopsy revealed a spindle cell neoplasm negative for keratin markers, pointing toward a differential of sarcomatoid carcinoma, undifferentiated melanoma, and pleomorphic sarcoma. ^18^F-FDG PET/CT scan (**B–D**) demonstrated multifocal uptake (SUVmax 12.6) with expansion of the right femoral and popliteal veins. Repeat duplex ultrasound (**E–G**) showed a hypoechoic heterogeneous intraluminal mass, and though challenging to identify, an arterial waveform was indicative of a tumor. MRI (**H–K**) confirmed a fusiform intraluminal femoral venous mass, isointense to muscle on pre-contrast Dixon water-only imaging (**H**) with avid peripheral enhancement on postcontrast Dixon water-only imaging (**I, K**), and fluid signal heterogeneously hyperintense on STIR imaging (**J**). A biopsy of the femoral vein lesion revealed a high-grade spindle cell neoplasm with focal necrosis, high mitotic activity, and staining for SMSA and desmin positive, supporting the diagnosis of intravenous high-grade leiomyosarcoma with pulmonary metastases. Leiomyosarcoma is a rare malignant mesenchymal tumor that arises from smooth muscle cells. It rarely arises from the vasculature, accounting for 2% of all cases.^1^ Venous involvement, notably the inferior vena cava, is more common than arterial involvement.^1–3,7^ The tumor is aggressive, with a high risk of metastasis, most commonly to the lungs, and a 5-year survival rate of 30%–65%.^5,6,10^ Clinically, these tumors present with nonspecific symptoms such as palpable mass, limb edema, and vague pain, leading to misdiagnosis. Venous leiomyosarcoma can be mistaken for DVT, particularly in patients with recurrent thrombosis unresponsive to anticoagulation. Accurate diagnosis requires clinical, radiologic, and pathologic evaluation. Multimodality imaging plays a key role in evaluating disease extent and distinguishing tumor from thrombus.^4,8,9^ ^18^F-FDG PET can show increased uptake in nonneoplastic aseptic DVT due to endothelial and leukocyte activation, with intensity of uptake declining over time.^11^ ^18^F-FDG PET may be helpful in differentiating benign thrombus from various types of malignant tumor thrombus. Sharma et al^12^ reported a statistically significant difference in mean SUVmax between bland thrombus (mean 3.2, range 2.3–4.6) and malignant thrombus (mean 6.0, range 2.3–13.8), suggesting a cut point of 3.6 (sensitivity, 71%, specificity 90%). However, it is well known that some malignancies have variable ^18^F-FDG uptake, and bland thrombus can occasionally mimic a tumor with intense uptake, thus making interpretation more challenging.^13–15^ In leiomyosarcoma, ^18^F-FDG uptake ranges widely (SUVmax 2.8–26.1) such that some tumors may not be easily identified with PET alone.^16^ Intravascular leiomyosarcoma, such as in our case, can show high intensity uptake, whereas other reported cases have shown relatively low uptake.^17^ Given its rarity and clinical overlap with more common vascular conditions, a high index of suspicion and diagnostic workup with multimodality imaging are essential for accurate lesion characterization to avoid delays in management.
